# Fluorescence spectroscopy as an efficient tool for staging the degree of liver fibrosis: an *in vivo* comparison with MRI

**DOI:** 10.1038/s41598-018-29370-1

**Published:** 2018-07-20

**Authors:** Shaiju S. Nazeer, Ariya Saraswathy, Sachin J. Shenoy, Ramapurath S. Jayasree

**Affiliations:** 1 Division of Biophotonics and Imaging, Biomedical Technology Wing Sree Chitra Tirunal Institute for Medical Sciences & Technology, Poojappura, Thiruvananthapuram, 695 012 Kerala India; 2 Division of In Vivo Models and Testing, Biomedical Technology Wing Sree Chitra Tirunal Institute for Medical Sciences & Technology, Poojappura, Thiruvananthapuram, 695 012 Kerala India; 3 Present Address: Department of Pathology, University of Illinois at Chicago, Chicago, Illinois USA; 4 Present Address: Department of Physics, NSS College, Pandalam, Kerala India

## Abstract

The study utilizes autofluorescence spectroscopy (AFS) along with multivariate spectral analysis for differentiating various stages of hepatic fibrosis. AFS has recently emerged as an efficient tool for evaluating the variations in different endogenous flurophores. In this study, the potential of AFS for differentiating the stages of liver fibrosis is assessed and compared with the results of enzyme evaluation, histopathology and the most advanced diagnostic tool, MRI. Using a fiber optic probe, the emission profile of the flurophores such as flavin adenine dinucleotide (FAD), lipofuscin-like lipopigments (lipopigments), porphyrins and the variation in the total hemoglobin concentration are evaluated *in vivo* on liver fibrosis induced animal models adopting a minimally invasive technique. Significant difference (p < 0.05) in the level of these biomarkers was observed between different stages of liver fibrosis. Normal hepatic tissue could be distinguished from mild and moderate hepatic fibrosis with a sensitivity of 95 to 100% and specificity of 90 to 100% using multivariate spectral analysis. The results are favourable to consider this technique as a potential tool for diagnosing liver fibrosis at an early stage, which is monumental as it otherwise can lead to cirrhosis and liver failure.

## Introduction

Optical spectroscopic techniques such as Raman, infrared, fluorescence and diffuse reflectance have been widely used and established as reliable tools for disease diagnosis in the last two decades^1–18^. Among them, fluorescence spectroscopy has attained ample acceptance in the quantitative and qualitative analysis of biological molecules and tissues. Fluorescence spectroscopy assisted by organic or inorganic chemical systems is used to monitor biologically relevant metallic ions, carbohydrates and proteins^19–21^. Meanwhile, the label free method, autofluorescence spectroscopy (AFS) is also found to be useful for the analysis of endogenous fluorophores like amino acids, structural proteins, enzymes, vitamins, lipids and porphyrins of biological tissues or fluids for disease diagnosis. Variations in the concentration of these biochemicals at different stages are useful to discriminate and classify the severity of the diseases. In oncology, AFS is used in the diagnosis of malignancies associated with organs like brain, breast, cervix, colon, liver, prostate and oral cavity^4–14^. This is a fast and economically affordable technique and is also useful in the follow up of treatments associated with wound healing progression and identification of saliva stains from human skin^22,23^. AFS is also proved to be useful in evaluating biochemical alterations associated with diseases like atherosclerosis and tooth decay^24,25^. However, *in vivo* use of this technique for the diagnosis of different stages of liver fibrosis has not been evaluated so far.

Liver fibrosis is the excessive accumulation of extracellular matrix proteins. Advanced liver fibrosis can lead to complications like cirrhosis which is reported as one of the leading cause of death by disease. The primary reasons for the structural and biochemical changes of liver, associated with liver fibrosis are identified to be hepatitis virus infections, fatty liver and excess intake of intoxicants^26,27^. Hepatotoxicity evaluation, imaging using techniques like ultrasound sonography (USS), computed tomography (CT), magnetic resonance imaging (MRI) and histopathology of biopsies are the conventional and advanced methods used in the diagnosis of structural and biochemical changes associated with liver^28–35^. Transient elastography (FibroScan) is a new tool used to evaluate liver fibrosis by measuring liver stiffness in a non invasive, fast, and reproducible manner^36^. This works by measuring shear wave velocity using an ultrasound probe^37^.

Monitoring the levels of liver specific enzymes Alanine Transaminase (ALT) and Aspartate Transaminase (AST) is another common method to evaluate liver toxicity/damage^28,29^. However, the enzyme level gives an indication of hepatocellular injury and fails to provide any aetiology related information. In the clinical setting, volumetric analysis of liver damage is initially done by USS. But USS lacks exact morphometric details of liver damage. However, techniques like CT and MRI can overcome this problem to a certain extent. However, their limitations like high cost, non availability at primary health care centres, need for additional contrast agents and dedicated expert team make these techniques unacceptable among common people^30–34^.

Liver biopsy followed by histological evaluation is now considered as the best method to assess morphological damage occurred in liver tissue^35^. But, the histology judgement is based on focally collected specimens and cannot be considered as representative of the entire liver. Hence it is the need of the hour, to hunt for alternative techniques preferably *in vivo* methods for early diagnosis of liver associated diseases.

In this *in vivo* study, we adopt a minimally invasive technique to collect the AFS from rat liver in order to detect the spectral changes of fluorophores from different stages of liver fibrosis. Fluorophores, FAD, lipopigments and porphyrins have been studied here for the early diagnosis and staging of different phases of liver injury. Additionally, total hemoglobin concentration is also evaluated from the spectral filtering modulation (SFM) effect. Earlier, we have reported the staging of different phases of liver injury using the autofluroscence signal from the flurophores, collagen, Nicotinamide adenine dinucleotide (NADH) and FAD^38^. The study focussed on the changes in redox ratio using a higher energy excitation at 320 nm. In the current study, we looked for the changes in FAD, lipopigments, porphyrins and the total concentration of hemoglobin to improve the diagnostic accuracy from the previous one. AFS results are also validated with conventional gold standard methods like liver specific enzyme test, histopathology and advanced diagnosis tool like MR imaging for the confirmation of the results. The studies on exact differentiation between the groups and the validation of dataset using the fluorescence spectral data was carried out using principal component analysis - linear discriminant analysis (PCA-LDA). This study also attempts to evaluate the spectral changes during the reversal of moderate fibrosis to normal state, on stoppage of the intoxicant.

## Materials and Methods

### Animal model development

Male Wistar rats with average body weight of 250 g were used for the experiments. The study was approved by the Institute Animal Ethics Committee of Sree Chitra Tirunal Institute for Medical Sciences and Technology in accordance with the regulation of Committee for the Purpose of Control and Supervision of Experiments on Animals, India (No. B 2982011 IX, dated: 19-10-2011). Animals were housed in individually ventilated cages. The atmosphere maintained were; temperature in the range 20 to 24 °C, humidity 60 to 75% with artificial light for 12 hrs daily.

A total of 20 rats (5 rats in each group) grouped as control, mild fibrosis, moderate fibrosis and reversal (on stoppage of intoxicant) were used. The animal model for liver fibrosis was developed as described previously by Constandinou *et al*.^39^. Equal parts (1:1) of CCl_4_ and olive oil were injected intraperitoneally, twice weekly. Animals received 0.2 mL/100 g of the CCl_4_-olive oil mix for the initial two weeks and 0.1 ml/100 g for the subsequent weeks. Mild fibrosis model was treated for 6 weeks and moderate fibrosis model for 8 weeks. Control animals were also treated with identical volumes of olive oil. In the reversal group, the administration of CCl_4_: olive oil mixture was stopped after 8 weeks and the animals were followed up till 12 weeks. Spectral acquisition was carried out after 3 days of specified duration of intoxicant administration/stoppage. For spectral acquisition, animals were anesthetized using ketamine (70 mg/kg) and xylazine (5 mg/kg) mixture. A small incision was made on the ventral side of the animal for accessing the liver for *in vivo* spectral acquisition.

### Autofluorescence Spectroscopy

#### Instrumentation and spectral acquisition

The autofluorescence measurements were carried out using a Fluorolog-III spectrofluorimeter (JobinYvon Inc., USA), equipped with a 450 W Xenon arc lamp, double monochromators at excitation and emission side and a photomultiplier tube. A bifurcated Y type fiber optic probe of diameter, 1 cm was used for the *in vivo* measurements. One arm of this Y-type connector excites the liver while the fluorescence signal was collected through the other arm. Double grating monochromators at the excitation and emission side helps to reduce scattering effect. All spectra were acquired at an excitation wavelength of 410 nm. From each animal, twelve spectra were recorded from different areas of the liver.

#### Spectral processing and curve fitting analysis to obtain area under the curve

Spectral data points were extracted from the original spectra using the software Datamax^TM^. All spectra were normalized with respect to the maximum intensity in the wavelength region 500 ± 10 nm. Area under the specific peak was analysed using a MATLAB based curve fitting program developed in house. Peak position, intensity, and bandwidth (full width at half-maximum) were assessed using Gaussian spectral functions. The fitting program uses the Levenberg-Marquardt algorithm to find the true absolute minimum value of the sum of squared deviations (the value of chi-square) by an iterative process^40^. Fitting program provides the quantitative measures like exact peak position and area under the specific flurophore. One-way ANOVA on the intensity values and area under the curve (AUC) values was performed between all the sixty observations in each group in order to obtain statistical significance value using the software SPSS-17 (SPSS Inc., Chicago, Illinois).

#### Total hemoglobin concentration estimation from the spectral data

SFM effect on fluorescence spectra has been utilised to evaluate total hemoglobin concentration^41^. Total hemoglobin concentration of the tissue at different stages of the liver injury was estimated by comparing the ratio of fluorescence emission intensities at 500 and 570 nm. The extinction coefficients of oxygenated and deoxygenated hemoglobin at these wavelengths are equal. Therefore the ratio between these two intensities provides the total hemoglobin concentration.

#### Multivariate statistical analysis

Principal component analysis (PCA) is an unsupervised statistical technique for extracting key variables within a multidimensional data set by simplifying complex datasets. Loadings and scores of PCA properly explains the differences in multidimensional data set. Linear discriminant analysis (LDA) is a supervised classification technique that converts a set of observations into its predefined classes. LDA determines the discriminant function that maximizes the variances between groups while minimizing the variances between members of the same group^1,3,5^. In this study, we carried out PCA followed by LDA to classify the measured AF spectra. Normalized dataset (spectrum ranging from 440 to 750 nm with a data set of 310 intensities) was used to generate principal components (PCs) using the software SPSS. These PC scores were used for the development of LDA algorithms for multiclass classification.

A training dataset of 50 randomly selected spectra from each group was used for this purpose. As the first step, 200 spectra from four different groups were subjected to PCA and the significant principal components were extracted. Pair-wise discriminant analysis was carried out on these extracted significant principal components from the training set. Subsequently, validation test was performed on the dataset (10 randomly selected spectra in each group) to further assess the suitability of the training dataset. Discriminant function scores from validation dataset were inserted into the scatter plot of the training set for validation.

To get the sensitivity and specificity of the developed diagnostic algorithm, binary calculations were carried out on the results based on pairwise PCA-LDA scores. Receiver operating characteristic (ROC) curve provided performance level of the pairwise PCA–LDA model by varying the discrimination threshold levels^17,18^. Again for more confirmative evidences, the results were further represented in the form of confusion matrix. For this, PCA-LDA modelling in the form of confusion matrix was also carried out using all spectral dataset (60 spectra in each group).

Details of conventional clinical analysis used in this study for the evaluation of liver fibrosis such as hepatotoxicity evaluation, MRI and histopathological analysis are included in the Supplementary Information (Section S.1)

## Results and Discussions

### Spectral features

**Figure 1:**
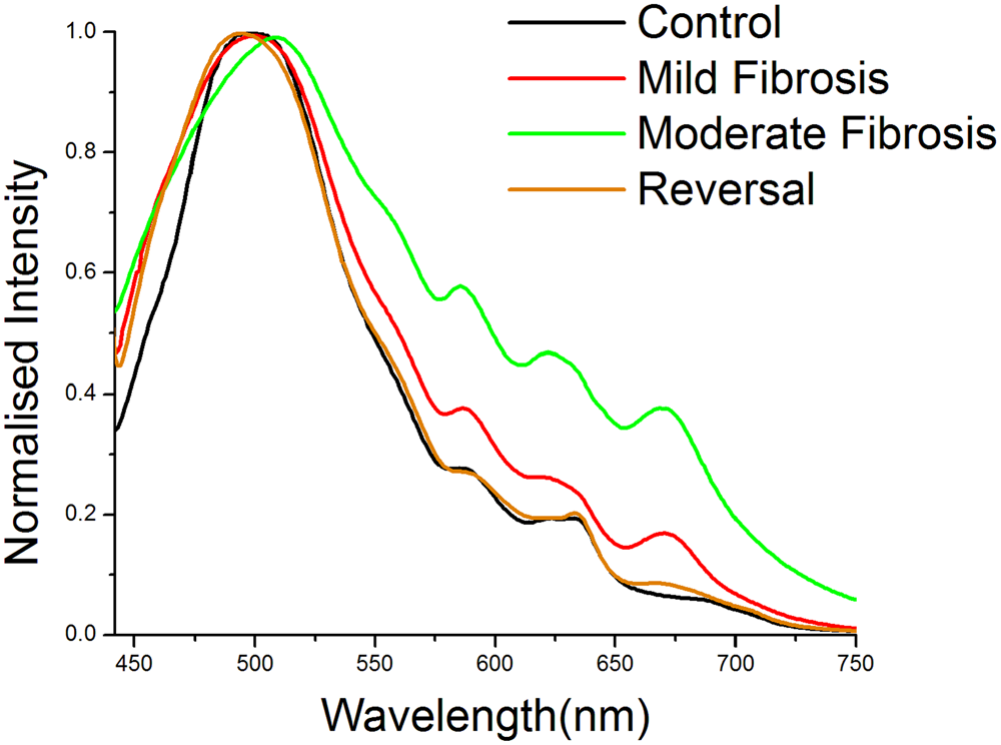
Normalized averaged fluorescence emission spectra of various degrees of intoxicant induced liver injury (410 nm ex).

**Figure 2:**
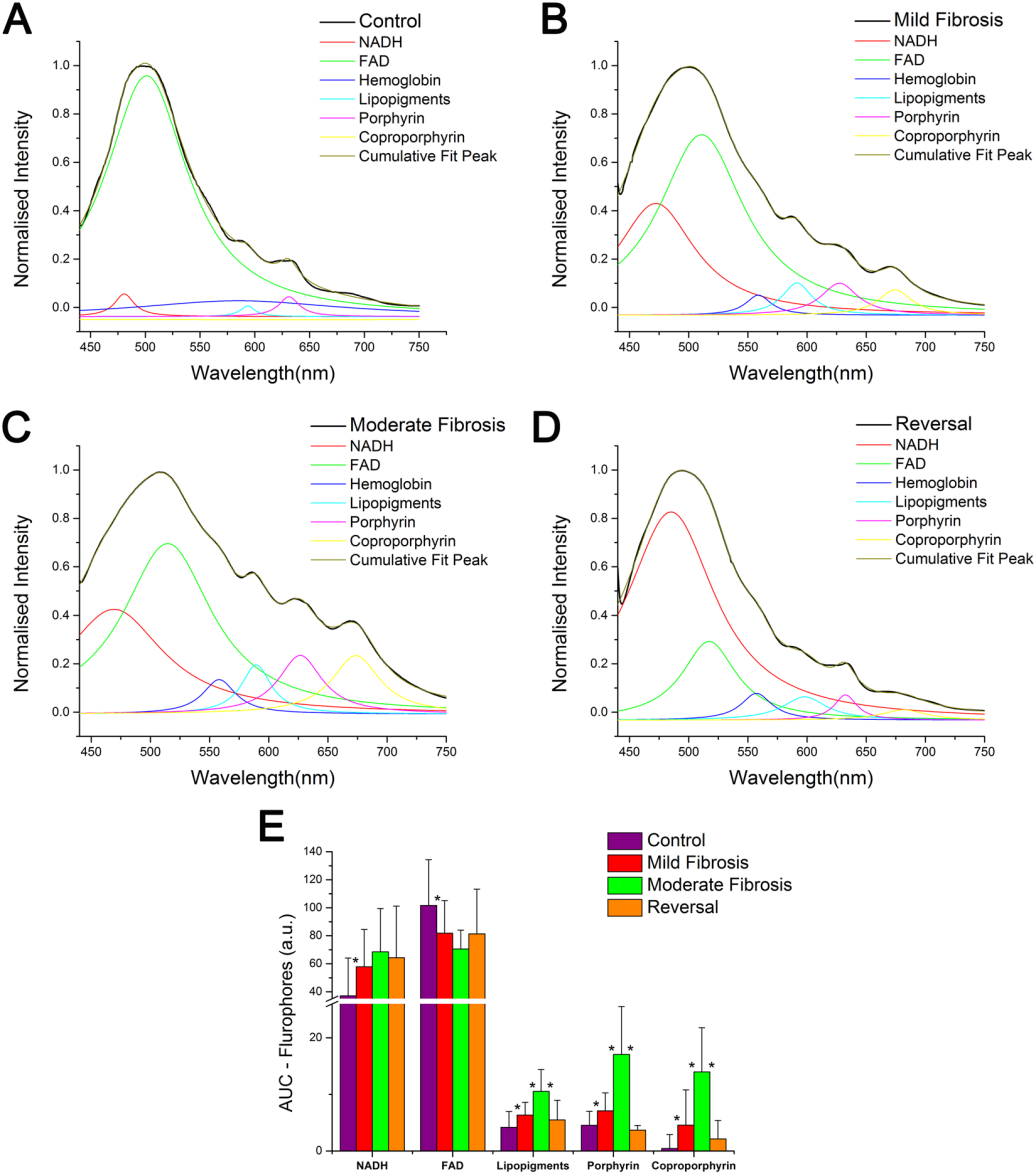
(**A–D**) Curve-fitting analysis on the averaged, and normalized autofluorescence spectra of various groups showing constituent peaks (**E**) Area under the emission peaks of NADH, FAD, lipopigments, porphyrin and coproporphyrin around 470, 500, 590, 630 and 670 nm respectively (Evaluated by Gaussian curve fitting). Data shown are Mean + SD. *Represents the statistically significant (p < 0.005) variation between the groups.

The normalized and averaged spectra of different groups of animals are shown in Fig. 1. The peaks around 500, 590, 630 and 670 nm are assigned to the emission of FAD, lipopigments, porphyrin and coproporphyrin respectively. The major emission peak is observed around 500 nm. The normalization of the whole spectral dataset was done based on the intensity of this peak. This peak is considerably red shifted in the case of moderate fibrosis. However, only a minor red shift is observed to this peak in the case of mild fibrosis. An increase in the intensity is observed for the other peaks, in both cases. The increase was significant in the case of moderate fibrosis, while it was less in the case of mild fibrosis. The spectra of control and the reversal were almost identical. There was no major change in the shape of the spectra among these two groups. Curve fitting analysis was performed to strengthen the spectral findings. It also showed prominent difference between the area under the curve of various fluorophores, among different groups (Fig. 2).

The lipopigments peak at 590 nm is more prominent and resolved in the case of mild and moderate fibrosis in comparison to the control^42,43^. Interestingly the lipopigments peak of reversal group is at par with that of the normal controls. Area under fluorescence peak also follows the same pattern for this fluorophore, with least area for control and reversal, and highest for moderate fibrosis group (Fig. 2). Phospholipids account for 50% of liver lipopigment lipids^44^. Metabolic changes have key role in the pathogenesis of both alcoholic and non-alcoholic liver disease. The increase in hepatic phospholipid level indicates the level of liver damage^45,46^. The result provides a clear indication of the change in the concentration of fluorophore. The reversal of liver fibrosis to normal is indicated by comparable range of lipopigments in the case of reversal group and control group on stoppage of intoxicant. Pair wise ANOVA performed on the peak intensity and area for lipopigment peak for the different pairs; control-mild fibrosis, mild fibrosis-moderate fibrosis and moderate fibrosis-reversal also shows significant difference (p < 0.005) in peak intensity and area for all pairs considered.

Peaks specific to the biomarker protoporphyrin IX (Pp IX) were observed around 630 and 690 nm^6–10^. Two weak and less resolved peaks were seen for Pp IX emission around 630 and 690 nm for the control animals whereas in case of mild and moderate fibrosis they were intense and well resolved (Fig. 1), indicating its high concentration in these stages. Presence of excess porphyrin in liver is known to be associated with an increased risk of liver cancer^47^. The 690 nm peak of control is blue shifted towards 670 nm in the case of mild and moderate fibrosis. The blue shift is to be attributed to the accumulation of endogenous fluorophore coproporphyrin III as a result of liver damage^10^. The intense peaks of moderate fibrosis reduce the intensity during reversal. The shift also disappears almost completely during reversal, with the 630 nm peak resembling that of the control. Area under fluorescence peaks also show a similar trend.

For biological specimens, fluorescence emission peak of FAD is expected to be around 500 nm. As the spectra were normalized with respect to this peak, change in peak intensity is not considered (Fig. 1). But normalization has no effect on the red shift associated with mild fibrosis and moderate fibrosis compared to control. A significant shift from the control (501 nm) is observed for moderate fibrosis (512 nm) whereas for mild fibrosis, it is seen at 505 nm. In the case of reversal, the peak resumes the position of control (502 nm). This significant red shift observed can be attributed to the structural changes identifiable using AFS, with further capability of differentiation between the stages. A blue shifted FAD emission has been reported for increased concentration of haemoglobin^8,41,48^ and hence it is assumed that red shift of FAD peak can be associated with decrease in hemoglobin concentration. Curve fitting analysis showed two constituent peaks around 470 nm and 500 nm under this broad peak centred at 500 nm. Significant difference in peak areas is observed only for control-mild fibrosis pair. As a result of normalisation, sum of area under 470 and 500 nm peaks are found to be nearly similar for all the groups.

### Estimation of total hemoglobin concentration

**Figure 3:**
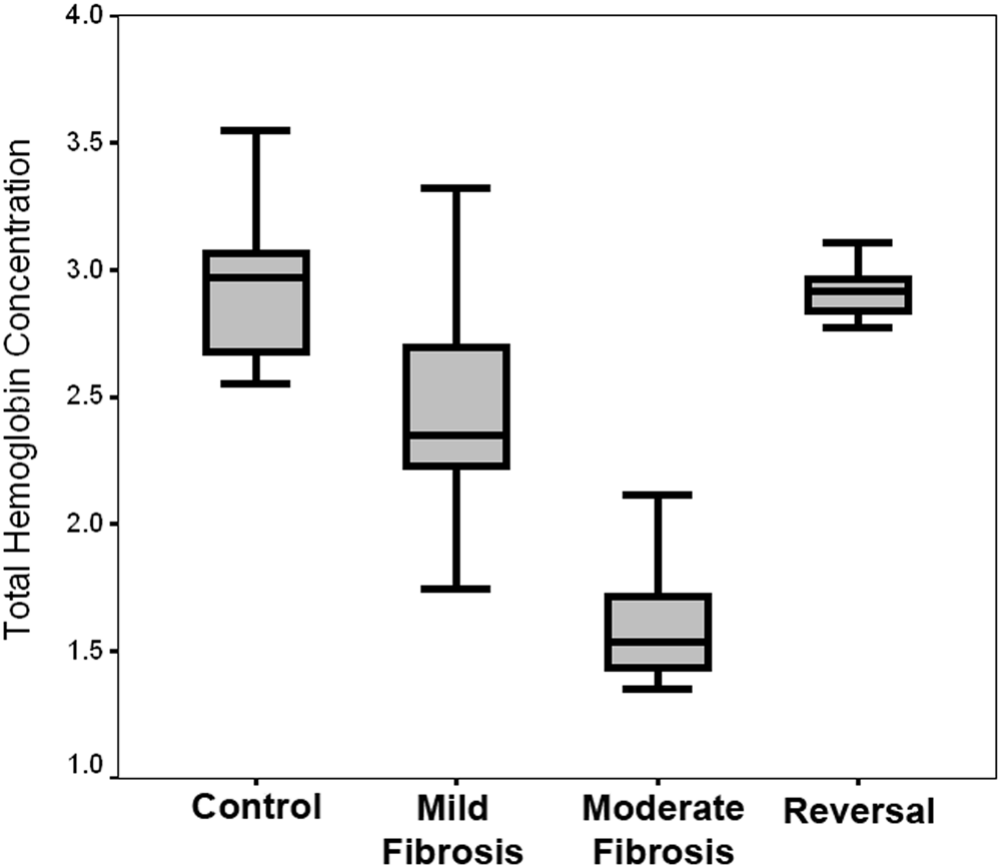
Box-and-Whisker plot represents variation in the total hemoglobin concentration of different grades of liver fibrosis. The middle line represents the median. The bottom and top of the box is 25^th^ and 75^th^ percentile of the data. The line extending from the top and bottom of the box represents the upper and lower extremes.

The peak corresponding to the spectral filtering modulation effect of hemoglobin absorption is observed at around 560 nm, in all the cases. Ratiometric method based on this spectral filtering modulation effect has been sparingly utilized to evaluate total hemoglobin concentration within a tissue^8,9,41,48^. The results of hemoglobin concentration evaluated using this method is represented as the Box-and-Whisker plot in Fig. 3. It shows remarkable decrease in total hemoglobin concentration in mild and moderate fibrosis compared to that of the normal liver. Decrease in hemoglobin is considered as a measure of fibrotic change that cause hepatocellular dysfunction and increased intra-hepatic resistance to blood flow leading to severe liver failure^49^. These findings justify the assumptions put forward on the basis of the red shifted FAD peak in the case of mild and moderate fibrosis. Reversal group of animals showed an increase in total hemoglobin concentration. This decrease or increase in total hemoglobin concentration is found to be statistically significant (p < 0.005) between different pairs.

### Multivariate data analysis

**Figure 4:**
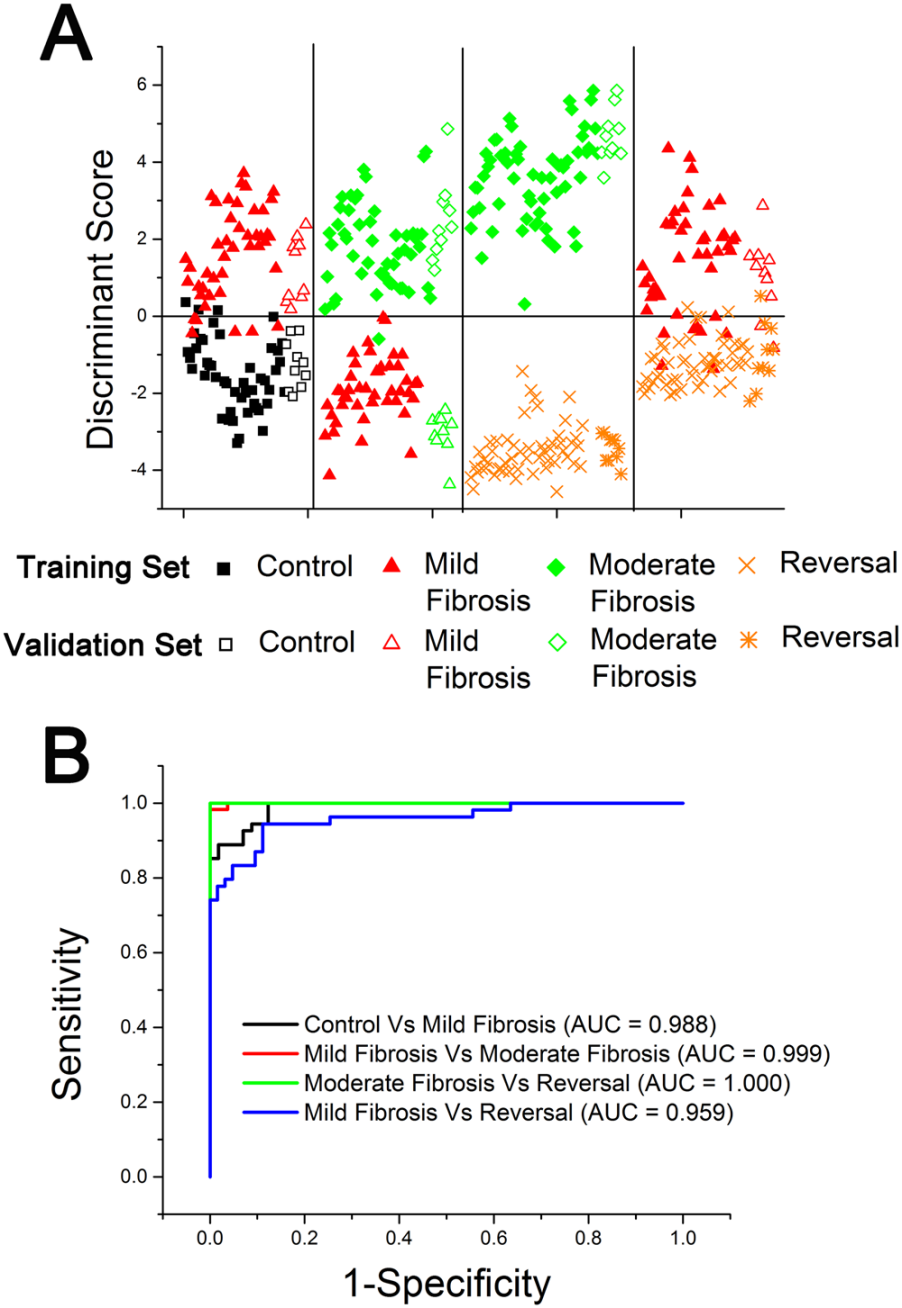
(**A**) Pairwise discriminant plot based on PCA-LDA for control-mild fibrosis, mild fibrosis-moderate fibrosis, moderate fibrosis-reversal and mild fibrosis-reversal pairs of liver lesions. Results of training dataset are represented by solid symbols and validation dataset are by open symbols. (**B**) The ROC curves of discrimination results obtained using PCA–LDA.

Based on the pair-wise discriminant function scores of PCA –LDA analysis, scatter plots were drawn and cut-off value was assigned to each pair (Fig. 4). The cut-off value of 0 gives good differentiation for all the pairs considered. Diagnostic accuracies such as sensitivity and specificity for each pairs were calculated by correlating the position of discriminant function scores for each lesion in the scatter plot. Sensitivities of 96%, 98%, 100% and 84% with corresponding specificities of 92%, 98%, 100% and 96% were obtained for pairs: control - mild fibrosis, mild fibrosis - moderate fibrosis, moderate fibrosis – reversal, and mild fibrosis - reversal respectively (Table 1).

**Table 1:** Overall diagnostic accuracies using multivariate analysis, PCA-LDA for different pairs consisting of 50 spectra in each group of the training set and 10 spectra of the validation set.

Lesion pairs	Control versus Mild Fibrosis		Mild Fibrosis versus Moderate Fibrosis		Moderate Fibrosis versus Reversal		Mild Fibrosis versus Reversal	
	Se (%)	Sp (%)	Se (%)	Sp (%)	Se (%)	Sp (%)	Se (%)	Sp (%)
Training set	96	92	98	98	100	100	84	96
Validation set	90	80	100	90	100	100	80	90
Overall	95	90	98.33	96.67	100	100	83	95

Se–Sensitivity.

Sp–Specificity.

In order to find the efficacy of the technique to differentiate between different grades of fibrosis among the groups, an algorithm based on PCA-LDA was developed. The developed algorithm for validation test could correctly classify the groups between control versus mild fibrosis, mild fibrosis versus moderate fibrosis, moderate fibrosis versus reversal and mild fibrosis versus reversal (Fig. 4). Accordingly it classified eight spectra as control and nine spectra as mild fibrosis from control - mild fibrosis pair (with two control spectra misclassified as mild fibrosis and one mild fibrosis spectra misclassified as control), nine spectra as mild fibrosis and ten spectra as moderate fibrosis from mild fibrosis - moderate fibrosis pair (with one mild fibrosis spectra misclassified as moderate fibrosis), the complete spectra in the moderate fibrosis-reversal pair, and 8 spectra as mild fibrosis and 9 spectra as reversal in the mild fibrosis-reversal pair (with 2 mild fibrosis spectra misclassified as reversal and one reversal spectra misclassified as mild fibrosis). This leads to sensitivity of 90%, 100%, 100% and 80% with specificity of 80%, 90%, 100% and 90% respectively for control versus mild fibrosis, mild fibrosis versus moderate fibrosis, moderate fibrosis versus reversal, and mild fibrosis versus reversal pairs in the validation test (Fig. 4). An overall diagnostic sensitivity of 83% to 100% and specificity of 90% to 100% was obtained in this study. This is better than our preliminary report based on the spectral variations of the fluorophores collagen, NADH, FAD and the derived optical redox ratio, using 320 nm excitation^38^.

To further evaluate the performance of PCA–LDA based dataset, the receiver operating characteristic (ROC) curves (Fig. 4B) were generated from the pairwise discriminant score by varying the discrimination threshold levels. Area under the ROC curve gave values 0.988, 0.999, 1.000 and 0.959 respectively for the pairs: control - mild fibrosis, mild fibrosis - moderate fibrosis, moderate fibrosis – reversal and mild fibrosis - reversal. A value greater than 0.60 obtained from area under the ROC curve is considered as a good classification^17,18^.

**Figure 5:**
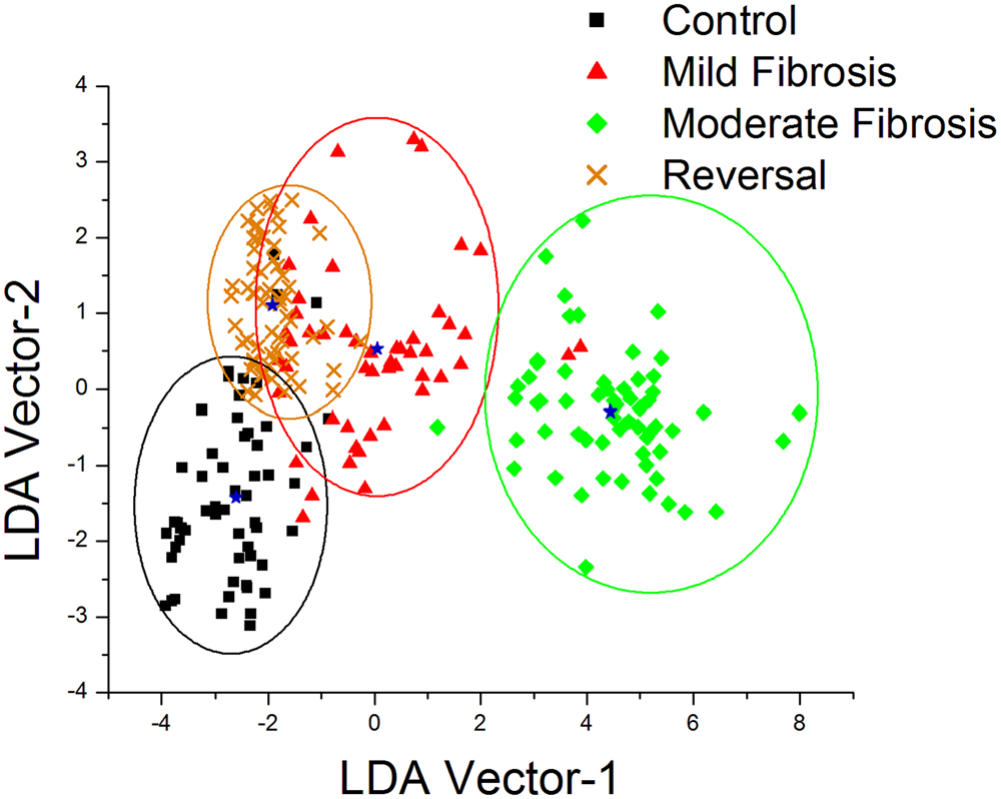
PCA-LDA score plots of the overall data using confusion matrix analysis. Star symbol represents the centroid of each group and the ellipsoids display the model confidence intervals for each class.

The classification efficiency was again checked using the confusion matrix analysis. Here, distinct clustering of data was observed with each class pooled within coherent point clouds. Moreover, data for each groups is mainly located within the classification model’s confidence intervals (double standard deviation, illustrated as ellipses) of the respective class^50^. In the confusion matrix, elements within the ellipse represent true positive predictions and ex-ellipsoidal elements indicate false positive predictions (Fig. 5). Averages of the discrimination value for each group represented as the centroids and are clearly distinguishable with substantial distance between each group. Here also, the classification among the different grades of fibrosis is very clearly manifested. Some overlapping is observed in the case of mild fibrosis and reversal. This is expected as the reversal of the moderate fibrosis to normal level has to pass through the mild fibrosis stage. It is again noted that the position of the reversal group lies between control and mild fibrosis indicating the chance of self-regeneration of liver on stoppage of intoxicants at appropriate time.

### Liver Function Test

**Figure 6:**
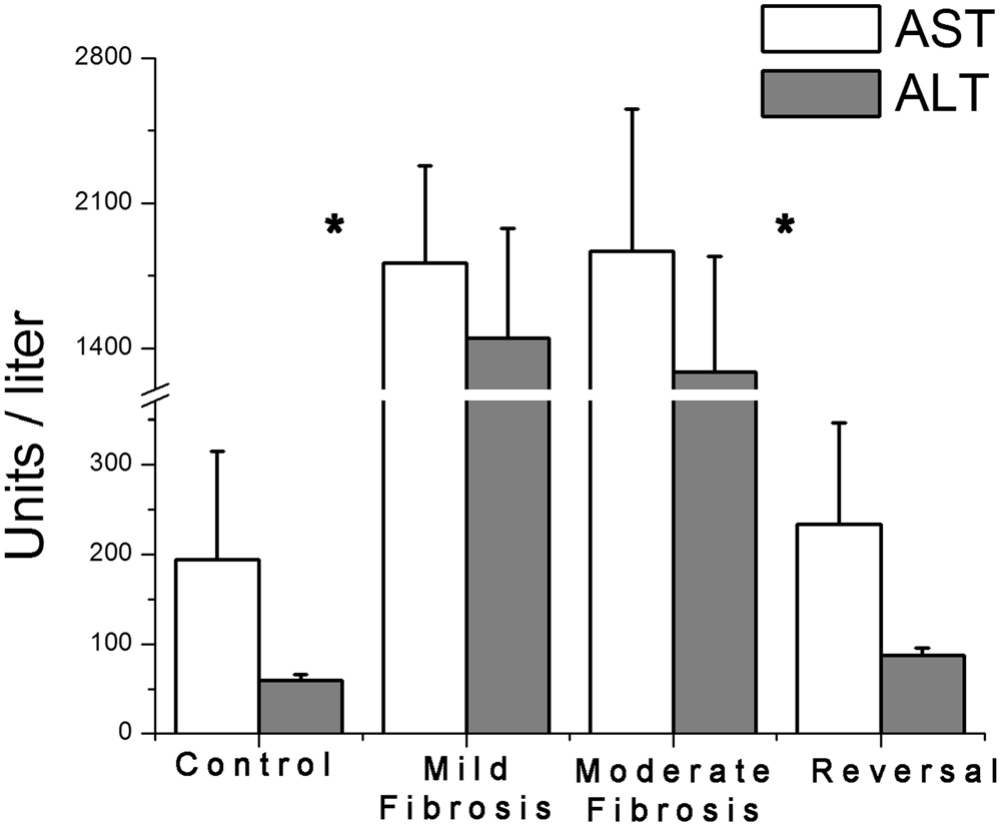
ALT and AST levels of control, mild fibrosis, moderate fibrosis and reversal groups. Elevated liver specific enzyme levels are observed for mild fibrosis and moderate fibrosis. In reversal, a decrease is observed matching with the level of control. The data represents mean ± SD. Significance (*p < 0.005) of differences between the groups.

Level of liver transaminases, ALT and AST were monitored periodically and are shown in Fig. 6. Ten to twelve fold increase in the level of these enzymes was observed in mild and moderate fibrosis compared to control and reversal group. Results show a definite difference between the pairs: control versus mild fibrosis and moderate fibrosis versus reversal with high statistical significance (p < 0.005). No significant difference in ALT (p = 0.939) and AST (p = 0.254) levels was observed between mild and moderate fibrosis. This proves the fact that though this test can differentiate between normal and injured liver it cannot differentiate between different grades of fibrosis.

### Magnetic Resonance Imaging

MRI is the most advanced imaging tool used to investigate the internal anatomy and physiology of living subjects. They exploit the phenomenon of nuclear magnetic resonance (NMR), whereby atomic nuclei exposed to a strong magnetic field absorbs and re-emit electromagnetic waves at a characteristic or ‘resonant’ frequency, which falls in the radio frequency (RF) range. This technique is extremely versatile, because of the wealth of information contained in the signal, regarding both the gross structural properties of the tissue and its biochemistry.

**Figure 7:**
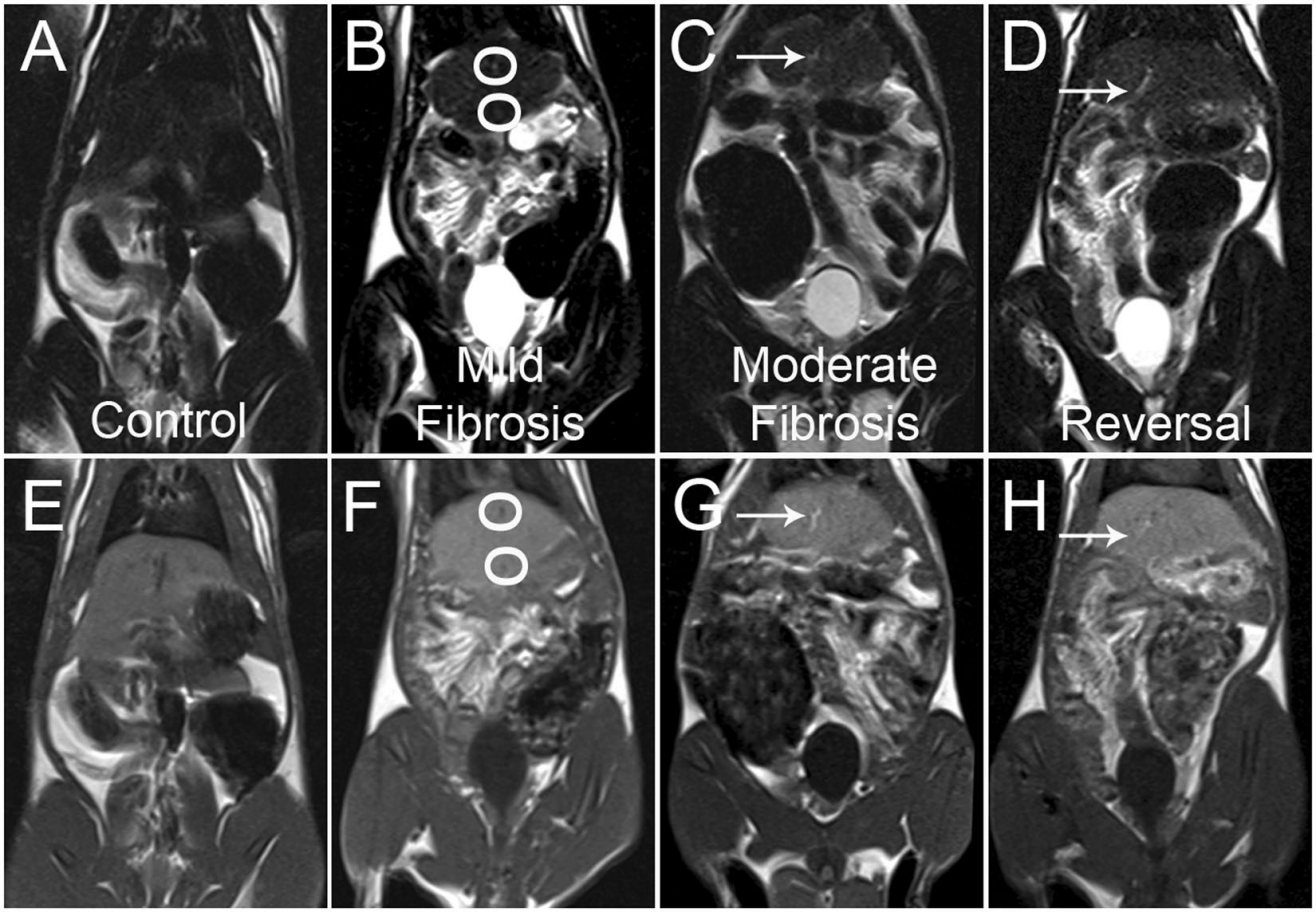
(**A**–**D**) T_2_ weighted and (**E**–**H**) T_1_ weighted MR images of control (A and E), mild fibrosis (B and F), moderate fibrosis (C and G) and reversal (D and H) animals. Circles mark the fibrosed areas while the arrows mark the compartmentalisation of the liver in the moderate fibrosis. Lace like structure is visible throughout the organ in T2 weighted image of fibrosis.

T_1_ and T_2_ weighted liver MR images of control, mild fibrosis, moderate fibrosis and reversal group of animals are shown in Fig. 7. T_1_ and T_2_ weighted imaging are standard protocols adopted for imaging in MRI. Information on the spin-lattice relaxation of the hydrogen protons of the tissue are reconstructed to get the T_1_ weighted images in MRI, whereas spin-spin relaxation, gives the T_2_ weighted images. In T_1_ weighted MR images, the mild fibrosis as well as the normal liver looks similar, without any major differences. Moderately fibrosed liver exhibits intermittent patches of hyper intensive areas^51^. But there is no remarkable visual delineation between the normal control and the mild fibrosis. T_2_ weighted image of mild fibrosis shows a lacelike pattern which is absent in the control. Characteristic morphological alterations such as surface nodularity, widening of fissures and relative enlargement of the lobes can be visualized in the MR image of the moderately fibrosed liver. Residual nodularity can be seen in the reversal group (Fig. 7D). The actual site of mild fibrosis/moderate fibrosis and the exact margins cannot be precisely distinguished from the MR image.

**Figure 8:**
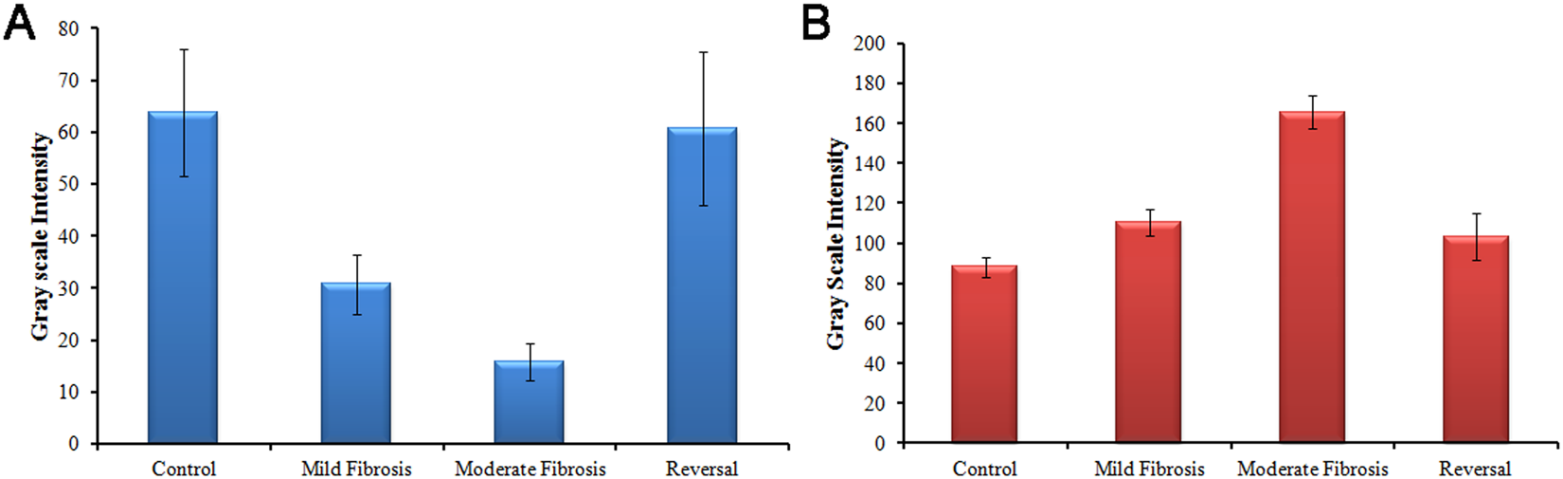
Contrast difference represented as gray scale intensity, evaluated from different groups of animals by image processing method (**A**) from T_2_ weighted and (**B**) from T_1_ weighted MR images. The results are the average of 20 regions of interest (ROIs) drawn on the liver.

The fibrosed areas are observed as low-signal-intensity reticulations on T_1_ weighted images and high-signal-intensity reticulations on T_2_ weighted images in the moderate fibrosis. These are indications of advanced fibrosis which is not visible in the images of control and mild fibrosis. The overall pixel intensity change is evaluated from the images and is shown in Fig. 8. As T_1_ weighted images provide an overall enhancement of pixel intensity, low intensity reticulations are not clearly visible whereas the visibility is better in the case of T_2_ weighted image. The results indicate that routine MR imaging is insufficient to provide biochemical details as well as the changes corresponding to various stages of liver fibrosis. It is reported that contrast enhanced MR imaging and advanced MRI techniques like MR spectroscopy and diffusion weighted imaging are preferred over conventional imaging for early diagnosis of liver fibrosis^33,34,52^. When externally administered contrast agents are required in the first case, advanced MRI sequences for measuring the apparent diffusion coefficient of water has to be run in the second case. In addition to the risk of injecting highly toxic gadolinium based contrast agents, these techniques involves high cost and extra time for running the additional MRI sequences, making it unreachable for common man.

### Histopathological evaluation

**Figure 9:**
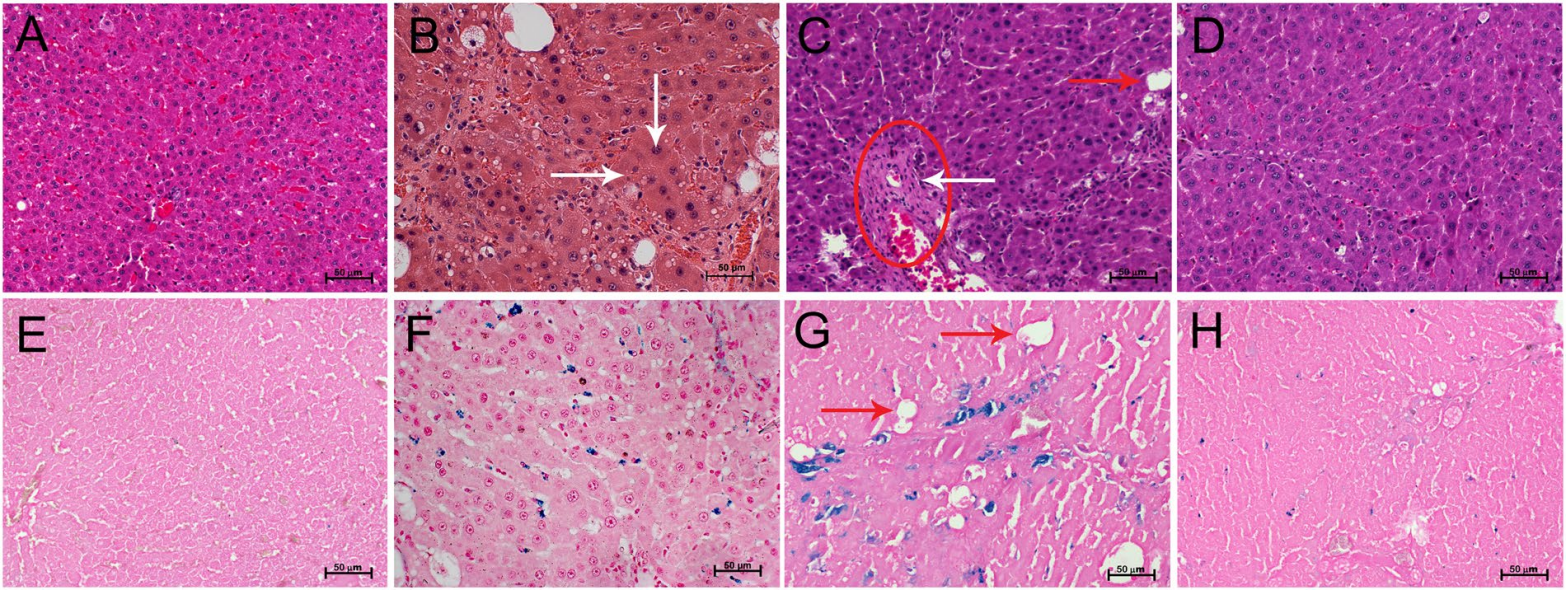
H&E and PB stained liver sections from (**A**,**E**) control, (**B**,**F**) mild fibrosis, (**C**,**G**) moderate fibrosis and (**D**,**H**) reversal of fibrosis animals. H&E stained section of mild fibrosis shows fatty changes (white arrows), ballooning of hepatocytes (red arrows) and nodule formation (circle) compared to control. These changes are prominent in moderate fibrosis than mild fibrosis. Liver section from reversal appears similar to the control liver. PB stained section of mild fibrosis, moderate fibrosis and reversal showing presence of iron (blue spots). Iron content is highest in moderate fibrosis and lowest in reversal.

Hematoxylin and eosin (H&E) stained sections of mildly fibrosed liver tissue shows increased inflammatory cell infiltration, ballooning of hepatocytes with infiltration of mononuclear cells, fatty changes and focal centrilobular necrosis compared with normal controls (Fig. 9). Increased parenchymal damage compared to mild fibrosis is also observed in moderate fibrosis. Reversal group shows pathological pattern similar to controls with some degree of centrilobular necrosis, inflammation and ballooning of hepatocytes.

Presence of iron as observed under Prussian Blue (PB) staining is attributed to iron overload, typically observed in liver damage^53^. The iron content is maximum in moderate fibrosis and minimum in reversal group (Fig. 9). Even though histological study provides good results, it cannot be considered as the representative of the entire organ, which is often based on the assessment from a biopsy sample.

Another promising new tool to diagnose liver fibrosis is transient elastography (FibroScan) which is a non invasive technique to measure the stiffness. As the fibrosis is progressed from mild to severe, an increase in liver stiffness value has been observed in the Transient elastography studies^36,54^. Though we could not compare the results of the present study with this technique, the technical limitation of minimally invasive nature of the currently proposed fluorescence spectroscopic technique is considered as one of the major limitations. However, with the use of powerful light sources and making suitable changes in the design of the fiber optic probe, this could be addressed in future.

## Conclusion

The focus of this study was on the classification of the grades of liver fibrosis using an economically viable and easy to handle method of AFS. Conventional diagnosis techniques like liver function test, MRI and histopathology were also carried out on experimental animals for evaluating liver fibrosis. This method for spectral evaluation indicates changes in flurophores like lipopigments, porphyrin and coproporphyrin and, chromophore hemoglobin during different stages of liver fibrosis. These biomarkers are known to play a major role in the biochemical and structural damage to the liver due to continuous and severe intoxication. Different stages of liver fibrosis induced by the use and discontinuation (reversal) of intoxicants were classified using AFS, assisted with multivariate data analysis method PCA-LDA. Comparable results of control and reversed stage indicate that stoppage of chronic alcohol or other intoxicant abuse at appropriate stage could repair the damaged liver. Method developed based on AFS is promising and may be evaluated in a clinical trial to distinguish patients with different stages of hepatic fibrosis, in future. An *in vivo* evaluation of lipopigments, porphyrins and the total hemoglobin concentration within liver tissues using AFS has not been reported so far. As a preliminary effort, the study proves the concept though it poses certain limitations, the major one being the demand of incision on the animal for accessing the liver. Development of dedicated systems with skin penetrating laser sources to collect the spectra without the need of an incision would address this issue and has the potential to replace currently adopted high cost and time consuming techniques.

## Electronic supplementary material

Supplementary information
