# Supplementary material for: Fluorescence spectroscopy as an efficient tool for staging the degree of liver fibrosis: an in vivo comparison with MRI
Source: Sci Rep. 2018 Jul 20;8:10967. doi: 10.1038/s41598-018-29370-1 (PMC6054616; doi:10.1038/s41598-018-29370-1)
Supplement: Supplementary file 1 — Supplementary information [file 41598_2018_29370_MOESM1_ESM.pdf]

Fluorescence spectroscopy as an efficient tool for staging the degree of liver fibrosis: an *in vivo* comparison with MRI

Shaiju S Nazeer[1#], Ariya Saraswathy[1+], Sachin J Shenoy[2], Ramapurath S Jayasree*[1]

[1]Division of Biophotonics and Imaging,
[2] Division *of In Vivo* Models and Testing,
Biomedical Technology Wing
Sree Chitra Tirunal Institute for Medical Sciences & Technology,
Poojappura, Thiruvananthapuram - 695 012, Kerala, India.

*Correspondence to: jayashreemenon@gmail.com

Current affiliations:
[#]Department of Pathology, College of Medicine Research Building (COMRB), University of Illinois at Chicago, Chicago, Illinois, USA.
[+]Department of Physics, NSS College, Pandalam, Kerala, India

Table of contents

1. Conventional Clinical diagnosis

2. References

Conventional Clinical analysis

Hepatotoxicity evaluation

Hepatotoxicity due to CCl_4 administration was assessed periodically by monitoring the level of liver specific enzymes in the blood, AST and ALT. For this, blood samples were collected from the rats by cannulating the jugular vein. Serum extracted from the blood samples were subjected to AST and ALT activity assessment by Reitman and Frankel method[1]. One-way analysis of variance (ANOVA) was carried out to find the difference between various groups.

Magnetic Resonance Imaging

In vivo MRI was performed on a 1.5 Tesla clinical MRI scanner (MAGNETOM Avento Tim, Siemens, Munich, Germany) using a 12 channel head coil. Longitudinal (T_1) and transverse (T_2) weighted imaging were performed with multisection and T_1-weighted TSE sequence (TE 11 ms; TR 468 ms) and T_2-weighted TSE sequence (TR 5780 ms; TE 125 ms) with a field of view of 98 X 140 mm; slice thickness 3 mm and flip angle 90 degree. All animals were anaesthetized using ketamine and xylazine mixture prior to the MRI scan.

Histopathological Analysis

Animals were sacrificed immediately after spectral acquisition and the liver was excised. The freshly excised tissues were fixed in 10% neutral formalin buffer. Formalin fixed specimens were subjected to microtome sectioning. Conventional histochemical staining was performed on these sections using hematoxylin and eosin (H&E) for morphological analysis. Pearls' Prussian blue (PB) staining was also carried out to assess the presence of iron in the tissue. Stained tissue sections were mounted on a bright field microscope (Leica DM IRB, Germany) and images were acquired at 20Xmagnification.

2. References

1. Reitman, S. & Frankel, S. A colorimetric method for the determination of serum glutamic oxalacetic and glutamic pyruvic transaminases. *American Journal of Clinical Pathology* **28**, 56-63 (1957).
